# Learnable Convolutional Attention Network for Unsupervised Knowledge Graph Entity Alignment

**DOI:** 10.3390/e27090924

**Published:** 2025-09-03

**Authors:** Weishan Cai, Wenjun Ma

**Affiliations:** 1 School of Computer Science, Guangdong University of Education, Guangzhou 510303, China; caiws@m.scnu.edu.cn; 2 Aberdeen Institute of Data Science and Artificial Intelligence, South China Normal University, Foshan 528225, China

**Keywords:** entity alignment, Knowledge Graphs, contrastive learning, learnable convolutional network, unsupervised learning

## Abstract

The success of current entity alignment (EA) tasks largely depends on the supervision information provided by labeled data. Considering the cost of labeled data, most supervised methods are challenging to apply in practical scenarios. Therefore, an increasing number of works based on contrastive learning, active learning, or other deep learning techniques have been developed, to solve the performance bottleneck caused by the lack of labeled data. However, existing unsupervised EA methods still face certain limitations; either their modeling complexity is high or they fail to balance the effectiveness and practicality of alignment. To overcome these issues, we propose a learnable convolutional attention network for unsupervised entity alignment, named LCA-UEA. Specifically, LCA-UEA performs convolution operations before the attention mechanism, ensuring the acquisition of structural information and avoiding the superposition of redundant information. Then, to efficiently filter out invalid neighborhood information of aligned entities, LCA-UEA designs a relation structure reconstruction method based on potential matching relations, thereby enhancing the usability and scalability of the EA method. Notably, a similarity function based on consistency is proposed to better measure the similarity of candidate entity pairs. Finally, we conducted extensive experiments on three datasets of different sizes and types (cross-lingual and monolingual) to verify the superiority of LCA-UEA. Experimental results demonstrate that LCA-UEA significantly improved alignment accuracy, outperforming 25 supervised or unsupervised methods, and improving by 6.4% in Hits@1 over the best baseline in the best case.

## 1. Introduction

Knowledge graphs (KGs) have progressively emerged as a new method to manage massive information, with various domains such as question answering [1], recommendation systems [2], reasoning [3], and safety and security [4] investigating their potential applications. Despite the rapid expansion in data volume and application scope, KGs still fall short of providing sufficient knowledge to support downstream tasks due to their inherent limitations in coverage. Consequently, it is imperative to explore integration techniques for heterogeneous KGs. A core task in this context is entity alignment (EA), which focuses on identifying equivalent entities across different KGs, thereby enabling the seamless merging of multiple KGs.

Recent advances in representation learning techniques have significantly propelled the development of embedding-based EA methods. Traditional EA methods initially depend on known aligned entities (also called alignment seeds or pre-aligned entity pairs) as supervisory signals. These methods project entities from different KGs into a unified embedding space, learn entity embeddings, and then subsequently employ vector similarity functions to measure entity similarities and predict alignment results. Existing EA methods can be classified into two primary categories: relation-based methods and auxiliary-based methods. Relation-based EA methods are grounded in the assumption that aligned entities possess similar relation neighborhood structures. These methods leverage translation-based models (e.g., TransE [5]) or Graph Neural Networks (e.g., GCN [6], GAT [7]) to extract relation structural features of entities. Auxiliary-based methods incorporate additional entity information, such as attributes, attribute values, images, etc., to enhance the embedding learning process for entities. Nevertheless, these methods still encounter certain limitations in contemporary applications, which undermine their effectiveness and robustness when applied to real-world KGs.

Limitation 1: Most models employ high-order networks to aggregate neighborhood information, but their training costs increase substantially. Owing to the powerful structure learning capabilities, Graph Neural Networks (GNNs) have been widely adopted as encoders in numerous studies [8,9,10] to enhance the ability to capture structural information within KGs. For instance, RPR-RHGT [8] and RANM [9] introduce variant attention mechanisms that incorporate the relation heterogeneity of KGs into the computation of attention coefficients. PEEA [10] proposes a novel position encoding method that integrates both anchor links and relational information from a global perspective. However, these methods rely on GNN variants or multi-layer stacking, resulting in a sharp increase in the number of parameters in neural networks, a significant increase in model complexity, and a substantial decrease in training efficiency. In actual large-scale KG scenarios, this problem is particularly prominent. Therefore, balancing the effectiveness and complexity of models remains a key challenge in EA tasks.

Limitation 2: Auxiliary-based methods exhibit limitations in terms of model effectiveness, practicality, and modeling efficiency. Firstly, while the incorporation of auxiliary information aids in enhancing entity feature extraction, it also introduces additional noise. The auxiliary information associated with different KGs is often inconsistent, rendering the selection or identification of valid and consistent auxiliary information a challenging task. Secondly, real-world KGs do not always encompass auxiliary information such as attributes, attribute values, images, etc. The more auxiliary information a method relies on, the greater the data requirements for its application, thereby making the method more scenario-dependent. Thirdly, it is evident that the introduction of more auxiliary information inevitably leads to an increase in method complexity. Auxiliary-based methods [11,12,13,14] require more complex models to handle auxiliary information across KGs. This issue has garnered increasing attention recently; however, no effective solution has yet been proposed.

**Figure 1:**
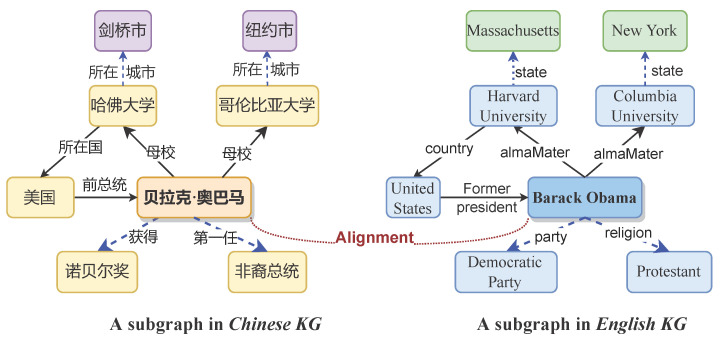
Illustration of the EA task.

To resolve the above limitations, we propose a learnable convolutional attention network for unsupervised entity alignment method known as LCA-UEA. For limitation 1, we abandon the use of complex network and introduce a novel GNN, LCAT [15], as the backbone network to model the graph structures of KGs. LCAT has the advantage of simplicity and outperforms existing benchmark GNNs in terms of robustness to input noise and network initialization. For limitation 2, we restrict model inputs to only partial relation information. As shown in Figure 1, the most aligned entity pairs, *a* and *b*, exhibit differing neighborhood structures; only a subset of their neighbors (connected by solid edges) are identical, while other neighbors (connected by dashed edges) introduce noise that interferes with alignment. To address this issue, we propose a novel reconstruction method for relation structures based on potential matching relations, which efficiently filters out effective neighbors of aligned entities. Specifically, inspired by RPR-RHGT [8], we incorporate only those triples that contribute positively to alignment into the LCAT model learning process. Additionally, we integrate entity-context embeddings as model inputs to enhance alignment performance without substantially increasing model complexity. These designs not only reduce the data flow into the encoder but also lower the model’s resource requirements.

In addition, we incorporate contrastive learning [16] to reduce the reliance on alignment seeds, thereby enhancing the practicality and robustness of our proposed method. Finally, we propose a novel similarity function for evaluating the similarity of aligned entities. This is motivated by the limitation of conventional similarity functions, which only account for the diversity of entity pairs based on their intrinsic features. Our experiments on three benchmark datasets demonstrated that LCA-UEA outperforms state-of-the-art EA methods. We further conducted comprehensive additional analyses to validate the effectiveness of our simplifying and learnable convolutional attention network. More specifically, we summarize our contributions as follows:

- A reconstruction method of relation structure based on potential matching relations is designed, which improves alignment accuracy while reducing the computational cost of model training.
- A learnable GNN model is introduced to learn entity features, which performs convolution operations before the attention mechanism, ensuring the acquisition of structural information while avoiding the superposition of redundant information.
- A novel similarity function based on consistency is proposed, enabling more accurate measurement of the similarity between candidate entity pairs.
- Extensive experiments conducted on three well-known benchmark datasets demonstrated that LCA-UEA not only significantly outperforms 25 state-of-the-art models but also exhibits strong scalability and robustness.

The remainder of this paper is organized as follows: Section 2 provides a concise overview of related work. Section 3 presents a formal definition of concepts relevant to our methods. Section 4 details our proposed method, LCA-UEA. Section 5 reports the experimental results and compares them with state-of-the-art alignment methods. Finally, Section 6 concludes the paper and outlines potential future research directions.

## 2. Related Work

Existing embedding-based EA methods can be classified into two categories based on whether they use a training set: (semi-)supervised methods and self-supervised or unsupervised methods. According to the graph structure modeling method, the former category can be further divided into two groups: translation-based methods and GNN-based methods. Also, GNN-based methods can be further subdivided into two subcategories: relation-based methods and auxiliary-based methods. In this section, we briefly review each of these related works.

### 2.1. EA Based on Translation Model

TransE [5] is an energy-based model that projects entities and relations of KGs into different spaces and finds suitable translations between them. Translation-based methods served as the primary methods in early EA research, leveraging TransE to encode the structural information of KGs. MTransE [17] was the earliest work in this domain, relying solely on the relational structures of KGs for alignment. Subsequent studies proposed various enhancements. For instance, JAPE [18] and AttrE [19] incorporate attribute modeling, MultiKE [20] integrates multi-view knowledge representation, and BootEA [21] optimizes performance through iterative strategies. Additionally, NAEA [22] combines TransE and GCN to enhance the extraction of entity features. However, translation-based methods often fall short of achieving the desired results, as the high heterogeneity of KGs renders it challenging to transform one KG into another using a linear mapping function akin to multilingual lexical space transformation.

### 2.2. EA Based on Relation Structures

Given that entities with similar neighborhood structures in different KGs are likely to be aligned, GNNs have emerged as the most popular solution for EA tasks. GNN-based methods use well-known GNN architectures (e.g., GCN, GAT) and their variants to extract neighborhood features of entities, which are utilized to make final alignment decisions. Relation-based methods are the most common methods, where the input consists solely of the relation structures of KGs, such as RDGCN [23], AliNet [24], and KAGNN [25]. These methods are highly practical, as they rely exclusively on the fundamental data (i.e., the relation structure) of KGs.

Some other works cleverly model both intra-graph and cross-graph information, such as Dual-AMN [26] and PEEA [10]. Here, the cross-graph information is constructed through Graph Matching Networks (GMNs). Moreover, some works also add modeling of heterogeneous information (e.g., relation edges), such as MRAEA [27], RPR-RHGT [8], and RANM [9]. These works propose or apply novel heterogeneous graph embedding methods to learn more effective entity representations.

Some researchers focus on the development of semi-supervised methods, such as MRAEA [27], RANM [9], and PEEA [10]. These approaches expand the training set through iterative strategies by generating new alignment seeds during the training phase. In addition to semi-supervised learning, other advanced techniques (e.g., active learning, deep reinforcement learning, adversarial learning) have been applied to enhance the efficiency and effectiveness of EA methods [28,29,30,31].

### 2.3. EA Based on Auxiliary Information

In addition to the relation structure, many studies add auxiliary information (e.g., attributes, entity descriptions, and images) into the entity encoding process. The relation structure represents the external relationships between entities, while the attribute structure (i.e., the attributes and attribute values of associated entities) represents the internal characteristics of entities. Attribute-based methods require simultaneous input of both the relation and attribute structures of KGs, such as HMAN [11], AttrGNN [12], MRAEA [27], MHNA [13], RoadEA [32], and EAMI [14].

There are also methods that leverage powerful pre-trained or language models to model descriptive information about entities, such as HMAN [11], SDEA [33], SKEA [34], and MMEA-cat [35]. Since some entities possess distinctive visual features (e.g., humans, objects, or animals) or have clear logo markings (e.g., companies, organizations, or associations), this characteristic is highly beneficial for alignment judgments. Therefore, some methods utilize image information as additional input, such as MMEA-cat [35], SKEA [34], and GEEA [31].

### 2.4. Self-Supervised or Unsupervised EA Methods

Most early EA methods are (semi-)supervised, utilizing alignment seeds during the training process. Despite the success of these methods, the critical requirement for labeled data remains a significant barrier to their practical application [36]. Tagging alignment seeds is inherently time-consuming and labor-intensive, prompting growing academic interest in self-supervised or unsupervised EA methods, which typically leverage external information to reduce reliance on labeled data.

Self-supervised learning involves automatically generating supervised signals from large-scale unlabeled data through auxiliary tasks (pretexts), and subsequently training models using supervised learning methods. For example, MultiKE [20] develops cross-KG inference techniques to enhance labeled data generation. EVA [37] leverages images as pivots to produce pseudo-labeled data. UPLR [38] computes the graph interaction divergence between entity pairs and adaptively selects confident samples from unlabeled data.

Unsupervised EA methods bypass the need for labeled data by constructing loss functions based on the intrinsic characteristics of data distributions. For example, ICLEA [36] and SelfKG [39] both employ contrastive learning to learn entity representations in an unsupervised manner. SEU [40] and UDCEA [41] reformulate EA tasks as assignment problems and propose novel unsupervised approaches that do not rely on neural networks. Specifically, they leverage machine translation and pre-trained language models to compute cross-lingual or cross-KG similarities across multi-view information (e.g., entity names, structures, and attributes), which are then integrated using global alignment strategies.

The supervised learning methods described above, particularly those leveraging auxiliary information, tend to achieve better results because alignment seeds and auxiliary information are generally more informative. However, these methods have the following limitations: (1) Auxiliary information often contains noise, necessitating customized pre-processing for most methods. (2) Incorporating auxiliary structures significantly increases model complexity, resulting in inefficient training. (3) Acquiring alignment seeds and auxiliary information is inherently time-consuming and labor-intensive for most KG applications, limiting the scalability of these methods. Existing unsupervised methods face similar challenges. Therefore, we propose LCA-UEA, which relies solely on basic semantic information and relation structures of entities. In this work, we avoid using complex neural network architectures, such as stacked or concatenated networks, heterogeneous GNNs, or GMNs. This design choice enhances the practicality and robustness of our method in real-world applications.

## 3. Preliminaries

Before starting the method description, we proceed to introduce the preliminary definitions.

**Definition** **1.** ***A knowledge graph*** *(KG) can be denoted as $G=(E,R,A,V,T_{R},T_{A})$, where E,R,A, and V, respectively, represent the entity set, relation set, attribute set and value set. $T_{R}\subseteq E\times R\times E$ denotes the relation structure, and $T_{A}\subseteq E\times A\times V$ denotes the attribute structure. However, we only focus on the relation structure in this paper, so a KG can be simplified to G=(E,R,T), where T denotes the set of relation triples.*

**Definition** **2.** ***Entity alignment*** *(EA) task aims to find matching entities with the same meaning from two KGs, $G^{1}=\left(E^{1},R^{1},T^{1}\right)$ and $G^{2}=\left(E^{2},R^{2},T^{2}\right)$. In practice, there is usually some pre-alignment involved in model training providing seed alignments. However, this paper focuses on unsupervised entity alignment models, and there is no involvement of seed alignments in our model training.*

For convenience, we put $G^{1}$ and $G^{2}$ together as a primal graph, G=(E,R,T), in the experiment, where $E=E^{1}\cup E^{2}$, $R=R^{1}\cup R^{2}$ and $T=T^{1}\cup T^{2}$. Formally, we denote embedding vectors using bold lowercase letters and embedding matrices using bold uppercase letters. In particular, $e_{i}^{t}$ denotes the embedding of the *i*-th object of the *t*-th type, and $E^{t}$ denotes the embedding matrix for all objects of the *t*-th type, where *t* refers to the type index and *i* refers to the object index within that type. Specific notations and their descriptions are summarized in Table 1.

## 4. Methodology

**Figure 2:**
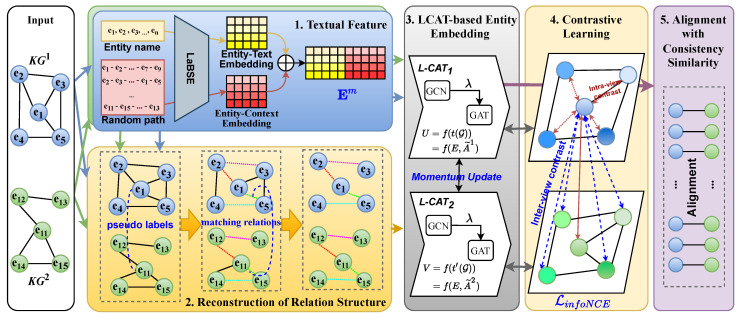
The overall architecture of LCA-UEA.

This section introduces the core work of this paper. Figure 2 depicts the architecture of LCA-UEA, which consists of five major components, as detailed below.

- The textual feature module extends traditional entity name embedding by introducing entity-context embedding, thereby enhancing the extraction of entity name information.
- The reconstruction of relation structure module aims to improve model efficiency and alignment performance by filtering out irrelevant neighborhood information for aligned entities during the data pre-processing stage.
- The LCAT-based neighborhood aggregator module employs a simple yet effective method to extract graph relation structures for entities.
- The contrastive learning module enables LCA-UEA to operate in an unsupervised method, eliminating the reliance on alignment seeds.
- The alignment with consistency similarity module proposes a novel consistency-based similarity function, which can measure the similarity of candidate entity pairs more effectively.

### 4.1. Textual Feature

Firstly, we construct the textual features of entities, which serve as the input for LCA-UEA training. In this work, we generate input embeddings by leveraging both entity-text information and entity-context semantics from KGs.

**Entity-Text Embedding.** The entity name serves as a fundamental form for entity recognition, encapsulating rich semantic information that is highly beneficial for alignment tasks. Therefore, many studies utilize pre-trained word embeddings to obtain entity name representations. Some works [23,40] employ Google Translate to convert non-English entity names into English counterparts. However, the entity embeddings generated by these methods are suboptimal due to translation errors and the out-of-vocabulary (OOV) problem encountered by existing pre-trained embeddings [42]. Multi-language embedding models are powerful tools for mapping text from different languages into a shared vector space. Among these models, LaBSE [43] is a language-agnostic BERT-based sentence embedding model developed by Google researchers. In this paper, we adopt LaBSE to encode entity name information. Without loss of generality, let $W\left(e_{i}\right)=\left(w_{1},w_{2},...,w_{n}\right)$ denote the entity name of $e_{i}\in E$, consisting of *n* words or characters. So we construct its name embedding using LaBSE as follows:

(1) $$e_{i}^{n}=f_{LaBSE}\left(W\left(e_{i}\right)\right),$$

where $f_{LaBSE}$ is a LaBSE encoder directly used to initialize the embeddings without additional fine-tuning.

**Entity-Context Embedding.** In the relation structure of KGs, most entities have associated entities within their context, which significantly aids in determining the similarity between aligned entities. Inspired by [42], we employ a random walk strategy to generate walk paths for each entity, thereby constructing sentences that encode dependency information from the entity-context. For any entity, $e_{i}$, a *k*-step random walk generates a walking path, $P_{e_{i}}=\left(e_{i},r_{1},e_{1},r_{2},...,r_{k},e_{k}\right)$. It is important to note that these walking paths include both entity nodes and relation edges. Different KGs not only have aligned entities but also aligned relation, and context sentences containing relation names better help the model distinguish different connection types. Incorporating relation edges into the long paths of entity-context is highly beneficial for uncovering the similarity between aligned entities. Subsequently, we also use the LaBSE model to learn features from these sentences and extract entity-context semantics, defined as follows:

(2) $$e_{i}^{c}=\underset{t\in [1,T]}{⊕}f_{LaBSE}\left(P_{e_{i}}\right),$$

where *T* indicates the number of random walk times, ⊕ indicates the superposition operation, and $e_{i}^{c}$ denotes the entity-context embedding of entity $e_{i}$. Given the randomness and uncertainty inherent in random walks, the results of a single simulation often have significant deviations and make it difficult to reflect the overall pattern. Therefore, multiple repeated random walks are adopted, and all simulation results are then superimposed and integrated to reduce the impact of random fluctuations. So, the superposition operation is to superimpose the output vectors of multiple random walk. In this paper, we choose to perform 10 random walks of the length 5 for each entity, that is, k=5 and T=10.

Finally, the multi-view embedding of entity $e_{i}\in E$ is calculated by concatenating two kinds of embeddings:

(3) $$e_{i}^{m}=e_{i}^{n}\left|\right|e_{i}^{c},$$

where || denotes the vector concatenation operation. The output of the textual feature layer is the feature matrix of all entities, $E^{m}=\left\{e_{1}^{m},e_{2}^{m},...,e_{n}^{m}\right\}$, where *n* represents the number of entities in *G*.

### 4.2. Reconstruction of Relation Structure

Most related works input the entire relation structure of KGs into convolutional networks. However, due to the diverse data sources of real-world KGs, the structures of different KGs are inherently heterogeneous. In other words, the neighborhoods of aligned entities are not fully matched in most cases. Some works address this issue by using random sampling to reconstruct the relation structure, which involves randomly selecting neighbor nodes of an entity. However, this method is prone to losing important neighbors. Following [8], we first generate matching relations using pseudo-labels, which are constructed through name embeddings. Then we propose a more effective method for restructuring the relation structure, which filters entity neighbors based on generated matching relations.

**Pseudo-Labels.** The relation matching method proposed in [8] relies on alignment seeds. However, in this paper, we focus on unsupervised learning, where alignment seeds are not available. To address this challenge, we generate pseudo-labels for new reliable alignment pairs, with the embedding of entity-text features constructed in the previous section. A simple method is to calculate the embedding distance for each entity pair and identify alignment pairs with distances smaller than a predefined threshold. However, this simplistic method may introduce errors.

To enhance the accuracy of the labeling results, we adopt bidirectional one-to-one alignment. Specifically, we first define a function for screening similar entity pairs:

(4) $$S\left(e_{j}^{2},E^{1}\right)=\left\{e_{i}^{1}|e_{i}^{1}\in E^{1},d\left(e_{i}^{1},e_{j}^{2}\right)>\gamma^{sim}\right\},$$

where $d\left(e_{i}^{1},e_{j}^{2}\right)=cosine\left(e_{i}^{1},e_{j}^{2}\right)$ is cosine similarity function, where a smaller value indicates a higher similarity between the entity pairs; $\gamma^{sim}$ is a pre-defined similarity threshold used to filter more reliable alignment pairs. Clearly, $D(e_{j}^{2},E^{1})$ is a filtering step, where each element has a similarity to $e_{j}^{2}$ exceeding $\gamma^{sim}$. Subsequently, we identify the set of pseudo-labels through one-to-one alignment.  

(5) $$\begin{array}{c} PL=\{\left(e_{i}^{1},e_{j}^{2}\right)|e_{i}^{1}=\underset{e_{p}^{1}\in D\left(e_{j}^{2},E^{1}\right)}{arg max}d(e_{p}^{1},e_{j}^{2}),e_{j}^{2}=\underset{e_{q}^{2}\in D\left(e_{i}^{1},E^{2}\right)}{arg max}d(e_{i}^{1},e_{q}^{2})\}. \end{array}$$

The key point of this operation is to select the most similar pseudo-aligned entity pairs from the filtered set.

**Relation Matching.** In this step, we establish similarity relations between the neighbors of each pseudo-label. For each $(e_{i}^{1},e_{j}^{2})\in PL$, we first compute the similarities between each pair of their respective neighbors. Next, we sort the pairs of neighbors with a similarity exceeding a predefined threshold, $\tau^{sim}$, in descending order and conduct one-to-one matching for the neighbors:

(6) $$\begin{array}{c} match\left(e_{i}^{1},e_{j}^{2}\right)=\left\{\left(e_{i^{\prime }}^{1},e_{j^{\prime }}^{2}\right)|\underset{e_{p}^{1}\in D\left(e_{j^{\prime }}^{2},N\left(e_{i}^{1}\right)\right)}{arg max}d(e_{p}^{1},e_{j^{\prime }}^{2}),\underset{e_{q}^{2}\in D\left(e_{i^{\prime }}^{1},N\left(e_{j}^{2}\right)\right)}{arg max}d(e_{i^{\prime }}^{1},e_{q}^{2})\right\}, \end{array}$$

where N(·) denotes the neighbors of an entity. Next, we derive the matching relation based on the following corresponding relationships:

(7) $$e_{i^{\prime }}^{1}↔e_{j^{\prime }}^{2}\Rightarrow \left(e_{i}^{1},r_{k}^{1},e_{i^{\prime }}^{1}\right)↔\left(e_{j}^{2},r_{p}^{2},e_{j^{\prime }}^{2}\right)\Rightarrow r_{k}^{1}↔r_{q}^{2},$$

where ⇒ indicates the matching relationship between two entities or two relations; $\left(e_{i}^{1},r_{k}^{1},e_{i^{\prime }}^{1}\right)$ is a relation triple corresponding to $e_{i}^{1}$ and its neighbor, $e_{i^{\prime }}^{1}$.

After that, we obtain all matching relations:

(8) $$R_{align}=\left\{\left(r_{k}^{1},r_{q}^{2}\right)|counter\left(\left(r_{k}^{1}↔r_{q}^{2}\right)\right)>\gamma^{r}\right\},$$

where counter(·) is employed to return the number of matches for a pair of relations, and $\gamma^{r}$ is a counting threshold. To minimize the impact of matching errors, we select only those matching relations whose counts exceed the threshold $\gamma^{r}$.

**Triples Reconstruction.** Finally, we use the matching relations to filter the triples in KGs, thereby achieving the reconstruction of the relation structure:

(9) $$T_{new}=\left\{\left(e_{i},r_{k},e_{j}\right)|\left(e_{i},r_{k},e_{j}\right)\in T,r_{k}\in R_{align}\right\}.$$

Algorithm 1 presents the procedure of this algorithm. It is important to emphasize that this algorithm serves only as a pre-processing step, meaning that it needs to be executed only once prior to model training. Thus, incorporating this module does not increase training complexity. Conversely, it reduces training complexity by decreasing the number of relation triples input into the EA model.

### 4.3. LCAT-Based Neighborhood Aggregator

The LCAT-based neighborhood aggregator is designed to update entity embeddings by performing message passing, leveraging the relation information from the KGs. This aggregator aggregates neighborhood information to the central node entity, which plays a crucial role in obtaining useful information for EA. As shown in Figure 2, LCA-UEA first applies different graph data augmentation techniques to construct two graph structures of KGs (this implementation will be elaborated on in the next subsection) and then utilizes LCAT as the backbone network to model these graph structures.
**Algorithm 1** Procedure of reconstruction of relation structure.**Input:** $G^{1}=\left(E^{1},R^{1},T^{1}\right)$, $G^{2}=\left(E^{2},R^{2},T^{2}\right)$, textual features $E^{m}$.**Output:** new relation triples $T_{new}$.  1:Set S←∅, PL←∅, $R_{align}\leftarrow \emptyset$, $T_{new}\leftarrow \emptyset$;▹ Generate pseudo-labels  2:**for** each $e_{i}^{1}\in E^{1}$ and $e_{j}^{1}\in E^{2}$ **do**  3:      **if** $d\left(e_{i}^{1},e_{j}^{2}\right)>\gamma^{sim}$ **then**  4:            Expand $S\leftarrow S\cup (e_{i}^{1},e_{j}^{2})$ ;  5:**for** each $(e_{i}^{1},e_{j}^{2})\in S$ **do**  6:      **if** $e_{i}^{1}=arg\max_{e_{p}^{1}\in D\left(e_{j}^{2},E^{1}\right)}d(e_{p}^{1},e_{j}^{2})$ and $e_{j}^{2}=arg\max_{e_{q}^{2}\in D\left(e_{i}^{1},E^{2}\right)}d(e_{i}^{1},e_{q}^{2})$ **then**  7:            Expand $PL\leftarrow PL\cup (e_{i}^{1},e_{j}^{2})$ ;▹ Generate matching relations  8:**for** $(e_{i}^{1},e_{j}^{2})\in PL$ and $\left(e_{i^{\prime }}^{1},e_{j^{\prime }}^{2}\right)\in match\left(e_{i}^{1},e_{j}^{2}\right)$ **do**  9:      **for** $\left(e_{i}^{1},r_{k}^{1},e_{i^{\prime }}^{1}\right)\in T^{1}$ and $\left(e_{j}^{2},r_{p}^{2},e_{j^{\prime }}^{2}\right)\in T^{2}$ **do**10:          $counter(\left(r_{k}^{1}↔r_{q}^{2}\right))++$ ;11:**for** $\left(r_{k}^{1},r_{q}^{2}\right)\in counter\left(\cdot \right)$ **do**12:      **if** $counter\left(\left(r_{k}^{1}↔r_{q}^{2}\right)\right)>\gamma^{r}$ **then**13:          Expand $R_{align}\leftarrow R_{align}\cup \left(r_{k}^{1},r_{q}^{2}\right)$ ;▹ Generate new relation structure14:**for** $\left(e_{i},r_{k},e_{i^{\prime }}\right)\in T^{1}\cup T^{2}$ **do**15:      **if** $r_{k}\in R_{align}$ **then**16:          Expand $T_{new}\leftarrow T_{new}\cup \left(e_{i},r_{k},e_{i^{\prime }}\right)$ ;

It is well known that a message-passing GNN layer generates node embeddings by collecting and aggregating information from its neighbors. This operation can be formally defined as follows:

(10) $${\tilde{e}}_{i}=\sum_{j\in N_{i}^{*}}\alpha_{ij}W_{1}e_{j},$$

where $W_{1}$ is a learnable matrix, $N_{i}^{*}$ denotes the set of neighbors of node *i* (including node *i* itself), $\alpha_{ij}\in \left[0,1\right]$ is the coefficient such that $\sum_{j}\alpha_{ij}=1$. Different GNN styles are determined based on the computation of $\alpha_{ij}$.

For example, GCN computes the average of messages by assigning the same coefficient, $\alpha_{ij}=1/\left|N_{i}^{*}\right|$, to each neighbor. In contrast to assigning a fixed coefficient, GAT calculates the attention coefficient for each neighbor as follows:

(11) $$\alpha_{ij}=\frac{exp(LeakyRelu\left(\psi \left(e_{i},e_{j}\right)\right))}{\sum_{k\in N_{i}^{*}}exp\left(LeakyRelu\left(\psi \left(e_{i},e_{k}\right)\right)\right)},$$

where $\psi \left(e_{i},e_{j}\right)={\vec{a}}^{T}[W_{1}e_{i}\left|\right|W_{1}e_{j}]$, $\vec{a}\in R^{2d}$ is the weight vector. As discussed in the Introduction, GCN or GAT have been widely used to obtain entity embeddings in many prior works. However, as pointed out in [44], these architectures exhibit certain limitations because their performance is highly data-dependent, as expected.

Therefore, we introduce the learnable convolutional attention network (LCAT) [15] to enhance the learning of embeddings for aligned entities. LCAT learns proper operations to apply in each layer, thereby integrating different layer types within the same GNN architecture. Relevant experiments also demonstrate that LCAT surpasses existing benchmark GNNs in terms of performance, network initialization, and robustness to input noise.

To exploit the advantages of both convolution and attention in the design of GNN architecture, LCAT extends the existing attention layer by introducing two learnable parameters to interpolate between GCN and GAT. This can be formulated as the following attention layer scores:

(12) $$\begin{array}{cc} {\tilde{e}}_{i} & =\frac{e_{i}+\lambda_{1}\sum_{k\in N_{i}^{*}}e_{k}}{1+\lambda_{1}\left|N_{i}^{*}\right|},\\ \psi (e_{i},e_{j}) & =\lambda_{2}\cdot ({\vec{a}}^{T}[W_{2}{\tilde{e}}_{i}\left|\right|W_{2}{\tilde{e}}_{j}]), \end{array}$$

where $\lambda_{1},\lambda_{2}\in \left[0,1\right]$ are the introduced learnable parameters. Here, $\lambda_{1}$ interpolates adding a mean-neighbor vector, while $\lambda_{2}$ interpolates between attention and no attention. So this formulation enables LCAT to interpolate between the GCN (when $\lambda_{2}=0$) and GAT (when $\lambda_{1}=0,\;\lambda_{2}=1$). Therefore, LCAT not only switches between existing layers but also learns the degree of attention required for each neighbor.

**Figure 3:**
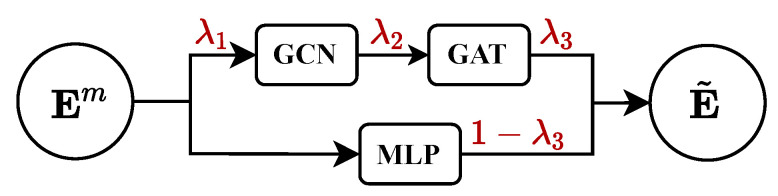
Intuitive illustration of the learnable parameters ($\lambda_{1},\lambda_{2},\lambda_{3}$).

In this paper, we adapt LCAT appropriately to make it more suitable for EA tasks. First, we employ a fully connected layer to model the textual features of entities, thereby enhancing the training of the model. Specifically, let $E=MLP(E^{m})$ serve as the input to the LCAT layer. By combining Equations (10)–(12), the output result of the LCAT layer can be obtained, denoted as $\tilde{E}$. Secondly, to capture the similarity of alignment in both textual features and neighborhood features, we introduce another learnable parameter to interpolate between the two, as follows:

(13) $$\tilde{E}=\lambda_{3}\cdot \tilde{E}+\left(1-\lambda_{3}\right)\cdot MLP\left(E^{m}\right),$$

where $\lambda_{3}\in \left[0,1\right]$ is the introduced learnable parameter, and $\tilde{E}$ is the output of our LCAT layer. Figure 3 provides an intuitive illustration of the above three learnable parameters.

### 4.4. Contrastive Learning

Contrastive learning enables the model to perceive structural differences by generating two distinct graph views without relying on labeled training data, thereby maximizing the consistency between the original KG and the augmented KG. Learning to distinguish positive and negative samples from two distributions is the key idea in contrastive learning. Inspired by the recent successful applications of unsupervised learning in EA tasks [16], we adhere to the common paradigm of graph contrastive learning, which seeks to maximize the consistency of representations across different views.

**Momentum update.** Specifically, we establish two encoders, the query encoder and the key encoder, each of which contains an LCAT network layer. The query encoder updates its parameters $\theta^{u}$ through gradient backpropagation in each training iteration. Meanwhile, the key encoder, which has the same structure as the query encoder, adopts a momentum update mechanism in each training iteration to maintain the consistency of negative samples. The parameters of the key encoder, namely $\theta^{v}$, are updated as follows:

(14) $$\theta^{v}=m\times \theta^{v}+\left(1-m\right)\times \theta^{u},$$

where m∈[0,1) is the momentum hyper-parameter.

**Contrastive learning.** Data augmentation in graph contrastive learning offers a complementary perspective by applying transformation operations (such as masking, denoising, and deletion) to the input graph. In this work, we implement graph data augmentation by masking some neighbors, which is one of the simplest methods. Specifically, we employ random graph augmentation on the relation structure using two distinct perturbation ratios, $\gamma_{1}$ and $\gamma_{2}$, which results in the generation of two complementary views of the KGs. Let ${\tilde{E}}^{k}$ and ${\tilde{E}}^{v}$ denote the output embedding matrix of the query encoder and key encoder, respectively. We adopt the InfoNCE loss function [16] to train the model, which aims to ensure that the node embeddings of each entity in the two views are consistent with each other while being distinguishable from the embeddings of other entities:

(15) $$L_{InfoNCE}=-\sum_{e_{i}\in E}log\frac{s({\tilde{e}}_{i}^{u},{\tilde{e}}_{i}^{v})}{s\left({\tilde{e}}_{i}^{u},{\tilde{e}}_{i}^{v}\right)+\sum_{k\neq i}s\left({\tilde{e}}_{i}^{u},{\tilde{e}}_{k}^{v}\right)+\sum_{k\neq i}s\left({\tilde{e}}_{i}^{u},{\tilde{e}}_{k}^{u}\right)},$$

where $s\left({\tilde{e}}_{i}^{u},{\tilde{e}}_{i}^{v}\right)=e^{{\tilde{e}}_{i}^{u}\cdot {\tilde{e}}_{i}^{v}/\tau }$, and τ is a temperature hyper-parameter. Under the guidance of the loss function, the model is optimized via backpropagation to learn entity embeddings.

### 4.5. Alignment with Consistency Similarity

After obtaining the final entity embeddings, we measure the similarities of candidate entity pairs. In real KGs, most entities have rather sparse neighborhood structures, while only a few entities are densely connected to others. As a result, the number of entities in real KGs follows a long-tailed distribution.

Most works use conventional similarity functions (e.g., cosine, Manhattan, Euclidean) to compute entity pair similarity, considering only the differences based on their own features. This results in two issues: first, the correlations between an entity and others are ignored; second, many one-to-many alignments appear in the results. To eliminate the impact of these discrepancies on the alignment, we reconstruct a similarity function based on consistency by introducing two local maxima (i.e., row maxima and column maxima of the similarity matrix) after the dot product operation, as implemented below:

(16) $$s\left(e_{s},e_{t}\right)=\left(\max_{e_{i}\in E^{2}}e_{s}\cdot e_{i}+\max_{e_{j}\in E^{1}}e_{t}\cdot e_{j}\right)/2-e_{s}\cdot e_{t}.$$

Here s(·,·) is essentially a distance (or negative similarity) measure; thus a smaller $s(e_{s},e_{t})$ means that $e_{s}$ and $e_{t}$ are each other’s top choices to a greater extent. This formulation effectively downranks scenarios in which, for example, $e_{s}$ is highly similar to another node compared to $e_{t}$ or vice versa, thereby mitigating the occurrence of one-to-many mappings. The final experiment also demonstrates that this function can eliminate the effects of the above differences and more effectively measure the similarity of entity pairs.

## 5. Experiments

### 5.1. Experiment Settings

**Datasets.** To fairly and comprehensively evaluate the performance of LCA-UEA, we conducted experiments on three extensive benchmark datasets, including two 15K standard datasets and a 100K large-scale dataset. The detailed statistics of the datasets are listed in Table 2.

- *DBP-15K* [18] is one of the most widely used datasets in the literature. It consists of three cross-lingual subsets derived from multi-lingual DBpedia: Chinese–English (${\mathrm{ZH-EN}}_{DBP}$), Japanese–English (${\mathrm{JA-EN}}_{DBP}$), and French–English (${\mathrm{FR-EN}}_{DBP}$). Each subset contains 15,000 aligned entity pairs but varies in the number of relation triples.
- *WK31-15K* [45] is designed to evaluate model performance on sparse and dense datasets. It comprises four subsets: ${\mathrm{EN-DE}}_{V1}$, ${\mathrm{EN-DE}}_{V2}$, ${\mathrm{EN-FR}}_{V1}$, and ${\mathrm{EN-FR}}_{V2}$. The V1 subsets represent sparse graphs obtained using the IDS algorithm, while the density of the V2 subsets is approximately twice that of the corresponding V1 subsets.
- *DWY-100K* [21] is a large-scale dataset suitable for evaluating the scalability of experimental models. It includes two monolingual KGs: DBpedia–Wikidata (DBP-WD) and DBpedia–YAGO3 (DBP-YG). Each KG contains 100,000 aligned entity pairs and nearly one million triples.

**Evaluation Metrics.** During the experimental evaluation, we used the similarity function defined in Equation (16) to rank candidate alignment pairs and adopted the following two standard evaluation metrics: Hits@*k* represents the proportion of correctly aligned pairs ranked among the top *k* candidates; the MRR (Mean Reciprocal Rank) is the average of the reciprocal ranks of the correct alignments. It is worth noting that higher Hits@*k* and MRR scores indicate better EA performance.

**Implementation Settings.** We followed the original data splits provided for *DBP-15K* [18] and *WK31-15K* [45]. For the unsupervised model, we allocated 10% of link pairs as the validation set and 70% as the test set. The dimension of input embeddings, batch size, number of epochs, momentum (*m*), and temperature τ were set to 768, 1024, 800, 0.999, and 0.08, respectively. For other hyper-parameters, we used the following configuration: $\gamma^{sim}=0.8$; $\gamma^{r}=5$; $\gamma_{1}=0.2$; and $\gamma_{2}=0.3$. Our proposed method was implemented using the Adam optimizer in the PyTorch 1.12.1 framework and experiments were conducted on a workstation equipped with an NVIDIA A5000 GPU located at South China Normal University in Guangzhou. The source code was made available on January 2025 at https://github.com/cwswork/SLU.

**Baselines.** To evaluate LCA-UEA, we compared it with the following three types of state-of-the-art EA methods, including both supervised and unsupervised methods.

- Supervised methods with pure relation structures: These methods are based on the original relation structures (i.e., triples): MTransE [17], BootEA [21], RDGCN [23], AliNet [24], RPR-RHGT [8], STEA [46], EMEA [47], PEEA [10], RANM [9], and KAGNN [25].
- Supervised methods with auxiliary information: These methods are based on both relation structure and some auxiliary information (e.g., attribute information, images), where JAPE [18], GCN-Align [48], MRAEA [27], AttrGNN [12], MHNA [13], and SDEA [33] use attribute or descriptive information and MMEA-cat [49] and GEEA [31] use image information.
- Unsupervised methods: These methods do not use training data, but some of them use some auxiliary information, including attribute information (MultiKE [20], AttrE [19], ICLEA [36], UDCEA [41]), descriptive information (ICLEA [36]), and images (EVA [37]). SEU [40] and SelfKG [39] only use the original relation structures.

As a reminder, our method relies solely on the structural information of KGs. For a relatively fair comparison, we replicated the UDCEA model by removing its attribute information. A similar case is the ICLEA method [36], which also incorporates description information.

### 5.2. Overall Results on DBP-15K and WK31-15K

In Table 3 and Table 4, we report the performance of LCA-UEA and the baselines on DBP-15K and WK31-15K. We categorize the baseline models into three groups, using horizontal lines for segmentation: supervised methods with pure relation structures, supervised methods with auxiliary information, and unsupervised methods.

**Comparison with supervised methods using pure relation structures.** Our proposed method was first compared with 10 supervised and relation-based methods, and it consistently achieved the best performance on all datasets except the ${\mathrm{JA-EN}}_{DBP}$ dataset. Specifically, compared with the second method, RANM [9], our method improved Hits@1 by 6.4% on ${\mathrm{FR-EN}}_{DBP}$ and by 6.1% on ${\mathrm{EN-FR}}_{V1}$. Since RANM considers the heterogeneous information of KGs and PEEA focuses on searching for one-to-one alignments, their training or alignment efficiency is lower than that of LCA-UEA. This shows that LCA-UEA remains highly competitive among this type of method, despite not performing as well as them on the ${\mathrm{JA-EN}}_{DBP}$ dataset. In addition, as an unsupervised method, LCA-UEA and some other unsupervised methods do not rely on labeled input data but still outperform these supervised methods. One of the main reasons for this is that the contrastive learning mechanism provides more positive samples (each entity serves as its own positive sample), and the InfoNCE loss function effectively extracts cross-KG entity information. In summary, LCA-UEA breaks the upper performance limit of relation-based EA methods and validates the effectiveness of its design.

**Comparison with supervised methods using auxiliary information.** Among the nine supervised methods with auxiliary information, the best performer was SDEA [33], which significantly outperformed our method. We attribute this to its effective integration of various types of information, particularly the attribute and description information of entities based on BERT [50]. Neither MMEA-cat [49] nor GEEA [31] considers entity name information but incorporate image information, while GEEA also considers attribute information. As shown in the experimental results, both methods exhibited lower performance compared to other baselines. This indicates that modeling entity names using pre-trained language models is more effective than relying on image-based methods. In conclusion, LCA-UEA still demonstrates better effectiveness and robustness due to its simpler model architecture and reduced reliance on input information.

**Compared with unsupervised methods.** From the experimental results alone, LCA-UEA achieved optimal performance on most datasets compared to the six unsupervised methods, but the performance improvement was not significant. However, we further observe the following: (1) Two translation-based methods, MultiKE [20] and AttrE [19], both consider attribute information but performed more generally. This indicates that translation-based models are less effective than GNNs for EA tasks. (2) The relation-based methods, SEU [40] and SelfKG [39], both achieved good results. However, SEU’s Sinkhorn operation requires $O(n^{2})$ algorithmic complexity, while SelfKG’s self-negative sampling strategy involves maintaining two negative sample queues. Compared to these two methods, LCA-UEA demonstrates better performance and modeling efficiency. (3) EVA [37] is a relatively early unsupervised model incorporating image information. Its performance was mediocre, and the difficulty in obtaining image information limits its applicability in real-world scenarios. (4) ICLEA [36] and UDCEA [41] consider both relation and attribute structures. ICLEA’s encoder is a multi-head GAT model, and UDCEA’s encoder is a multi-language Sentence-BERT. Since our LCA-UEA’s encoder consists of a single LCAT layer, the number of training parameters in the neural network is lower than that in the previous two methods. In summary, our LCA-UEA exhibits certain advantages over other unsupervised methods.

### 5.3. Overall Results on DWY100K

To verify the effectiveness of our method on a large-scale dataset, we report an end-to-end comparison of LCA-UEA with 16 baselines on the *DWY100K* dataset. As shown in Table 5, LCA-UEA first outperformed all other unsupervised methods and achieved the best performance across all metrics. Second, the Hits@1 scores of supervised methods on *DBP-WD* reached up to 99.3%, which is only 1% higher than LCA-UEA’s 98.3%. However, our LCA-UEA method still significantly outperformed all supervised methods on *DBP-YG*. Finally, while most baselines exhibited commendable performance, LCA-UEA’s Hits@1 on *DBP-YG* clearly reached 100.0%. This indicates that the monolingual setting effectively alleviates name bias and enhances the recognition of aligned entities. Overall, given that the size of *DWY-100K* is several times larger than that of *WK31-15K* and *DBP-15K*, this experiment demonstrated the excellent scalability and superiority of our method for larger real-world and monolingual KGs.

### 5.4. Ablation Experiments

In the previous section, we demonstrated the overall success of LCA-UEA. To validate the effectiveness of each component design in LCA-UEA, we conducted ablation studies using five variants of LCA-UEA on *DBP-15K*, and the results are shown in Table 6.

- *w/o ECE+RRS*: The modules for entity-context embedding and reconstruction of relation structure were removed;
- *w/o ECE*: The module for entity-context embedding was removed;
- *w Cosine*: The similarity function based on consistency was replaced with the cosine function;
- *w GAT*: The LCAT model was replaced with a simple GAT model;
- *w GCN+GAT*: The LCAT model was replaced with a stacked network of GCN+GAT.

As illustrated in Table 6, LCA-UEA achieved the best performance across most metrics and datasets. First, it can be observed that the Hits@1 of *w/o ECE* and *w/o ECE+RRS* degraded by 0.2–1.9% and 0.3–2.1%, respectively. The results confirm the effectiveness of the entity-context embedding, as it further extracts information about entity names compared to simply utilizing individual entity names. Although the effect of relation structure reconstruction was not significant, its key role was to reduce the amount of model training by removing some triples (those that had no effect on alignment) before training the model. Second, we tested the performance of LCA-UEA without using the new similarity function, meaning it relied on a universal cosine function to calculate entity similarity, which is also the case for most baselines. The results demonstrate that the similarity function based on consistency was significantly effective, bringing about an absolute improvement of 2.2–4.5% in Hits@1. Third, to analyze the effect of the LCAT model, we compared the performance of *w GAT*, *w GCN+GAT*, and LCA-UEA. The LCAT model effectively captured rich and subtle alignment information for the EA task.

### 5.5. Additional Analysis

In this section, we first investigate the stability of EA methods on datasets with varying densities. A sensitivity analysis was conducted on the hyper-parameters of graph data augmentation, specifically the perturbation rate $\gamma_{1}$, temperature τ, and momentum coefficient *m*, to evaluate their impact on the robustness of LCA-UEA.

**Effect of dataset sparsity.** The *WK31-15K* dataset contains four subsets with varying densities, where V1 represents a sparse dataset and V2 represents a dense one. Intuitively, EA methods tend to perform better on dense KGs because their entities possess richer neighborhood information. From Table 4, most supervised methods aligned with this intuition; their performance on V2 was significantly better than that on V1, particularly for AliNet, PEEA, and MRAEA. However, this trend did not hold for most unsupervised methods, as the performance difference between the V1 and V2 datasets was relatively small. This indicates that supervised methods, guided by alignment seeds, can train models to more effectively capture the similarity of aligned entities within neighborhoods. Unsupervised methods, in contrast, rely more heavily on entity-level information (e.g., entity names) during model training to infer alignments and thus do not demonstrate significant improvements in capturing neighborhood features. Therefore, enhancing the ability of GNN-based models to acquire neighborhood features under the unsupervised learning framework remains one of our key research directions.

**Figure 4:**
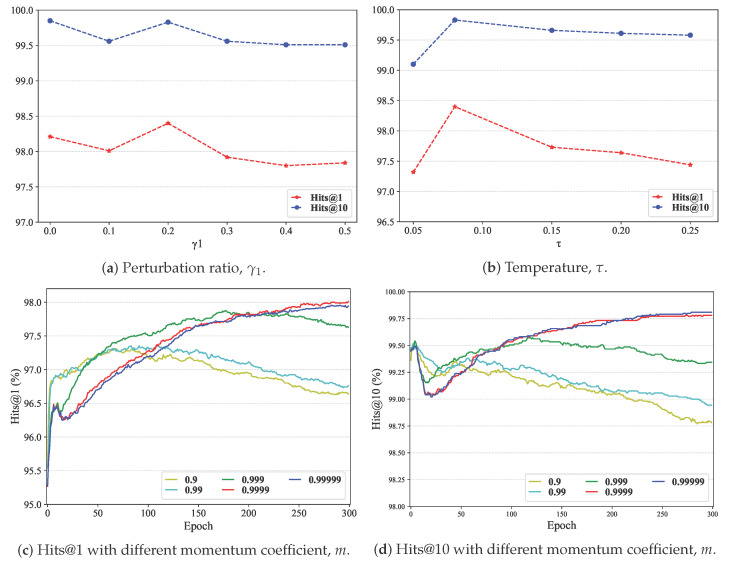
Performance comparison with different perturbation ratios, temperatures, and momentum coefficients on ${\mathrm{FR-EN}}_{DBP}$ dataset.

**Impact of perturbing ratio $\gamma_{1}$.** In graph data augmentation experiments, it is common to vary the perturbation rate in one view while keeping the other fixed. We set six perturbation ratios from 0.0 to 0.5; a zero ratio means that no disturbance is applied in one view. Clearly, higher ratios introduce more noise into the graph data. As shown in Figure 4a, LCA-UEA’s performance remained stable despite increasing perturbation. This shows that LCA-UEA is robust to such noise and that moderate noise can improve performance.

**Impact of temperature τ and momentum coefficients *m*.** Temperature and momentum are standard hyper-parameters in contrastive learning. The temperature τ controls the focus on difficult samples, while the momentum coefficient *m* stabilizes model updates [36]. We selected two sets of ensemble data based on prior work and show the results in Figure 4b–d. The results indicate that larger values of *m* lead to more stable performance, and τ=0.08 achieves a better balance, as shown in most studies. Overall, these experiments show that LCA-UEA is relatively insensitive to hyper-parameters, maintaining robustness during tuning.

## 6. Conclusions

Recent EA methods include both supervised and unsupervised approaches. However, these methods often face two main challenges: balancing effectiveness and efficiency, and managing increasing model complexity. To address these issues, we propose LCA-UEA, a novel unsupervised EA method that integrates five modules to improve alignment accuracy. We conducted extensive experiments on three diverse datasets to evaluate LCA-UEA. The results show that LCA-UEA outperforms several state-of-the-art supervised and unsupervised methods. We further analyze each module and find that our consistency-based similarity function significantly improves alignment performance. Moreover, experiments show that our redesigned relation structure module reduces complexity while improving performance. Additionally, we observe that GNN-based models in LCA-UEA do not significantly outperform those in some supervised methods. Therefore, our future work will focus on enhancing the ability of GNNs to capture neighborhood features in unsupervised settings.

## Figures and Tables

**Table 1:** Notations and descriptions.

Notation	Description
$E^{n}$	Text embedding matrix of entities.
$E^{c}$	Context embedding matrix of entities.
$E^{m}$	Output embedding matrix of the textual feature layer.
$\tilde{E}$	Output embedding matrix of the LCAT layer.
${\tilde{E}}^{u}$	Output embedding matrix of the query encoder.
${\tilde{E}}^{v}$	Output embedding matrix of the key encoder.
⊕	Superposition operation.
‖	Vector concatenation operation.
·	Dot product operation.

**Table 2:** Statistics of datasets.

Datasets		KGs	Entities	Rel.	Rel. Triples
*DBP-15K*	${\mathrm{JA-EN}}_{DBP}$	Japanese	65,744	2043	164,373
		English	95,680	2096	233,319
	${\mathrm{FR-EN}}_{DBP}$	French	66,858	1379	192,191
		English	105,889	2209	278,590
	${\mathrm{ZH-EN}}_{DBP}$	Chinese	66,469	2830	153,929
		English	98,125	2317	237,674
*WK31-15K*	${\mathrm{EN-DE}}_{V1}$	English	15,000	215	47,676
		German	15,000	131	50,419
	${\mathrm{EN-DE}}_{V2}$	English	15,000	169	84,867
		German	15,000	96	92,632
	${\mathrm{EN-FR}}_{V1}$	English	15,000	267	47,334
		French	15,000	210	40,864
	${\mathrm{EN-FR}}_{V2}$	English	15,000	193	96,318
		French	15,000	166	80,112
*DWY-100K*	DBP-WD	DBpedia	100,000	330	463,294
		Wikidata	100,000	220	448,774
	DBP-YG	DBpedia	100,000	302	428,952
		YAGO3	100,000	21	502,563

**Table 3:** Comparative results of LCA-UEA against 23 baselines on *DBP-15K*. Underline indicates the best results for the first two categories, while **bold** marks the best results for the unsupervised methods.

Datasets	${\mathrm{ZH-EN}}_{\mathrm{DBP}}$			${\mathrm{JA-EN}}_{\mathrm{DBP}}$			${\mathrm{FR-EN}}_{\mathrm{DBP}}$		
**Models**	**Hits@1**	**Hits@10**	**MRR**	**Hits@1**	**Hits@10**	**MRR**	**Hits@1**	**Hits@10**	**MRR**
MTransE [17]	30.8	61.4	36.4	27.9	57.5	34.9	24.4	55.6	33.5
BootEA [21]	62.9	84.8	70.3	62.2	85.4	70.1	65.3	87.4	73.1
RDGCN ‡ [23]	70.8	84.6	74.9	76.7	89.5	81.2	88.6	95.7	90.8
AliNet [24]	53.9	82.6	62.8	54.9	83.1	64.5	55.2	85.2	65.7
EMEA * [47]	78.2	93.3	84.2	77.1	95.0	83.7	80.1	96.6	86.3
RPR-RHGT ‡ [8]	69.3	86.9	75.4	88.6	95.5	91.2	88.9	97.0	91.0
PEEA *† [10]	76.1	91.5	81.6	77.2	92.5	82.1	80.6	94.5	85.8
RANM ‡ [9]	77.6	88.1	81.3	90.5	95.2	92.3	90.9	95.8	92.7
KAGNN ‡ [25]	73.6	87.3	78.6	79.4	91.1	83.7	92.0	97.6	94.1
JAPE [18]	41.2	74.5	49.0	36.3	68.5	47.6	32.4	66.7	43.0
GCN-Align [48]	41.3	74.4	54.9	39.9	74.5	54.6	37.3	74.5	53.2
MRAEA [27]	75.7	92.9	82.7	75.7	93.3	82.6	78.0	94.8	84.9
AttrGNN † [12]	79.6	92.9	84.5	78.3	92.0	83.4	91.8	97.7	91.0
MHNA * [13]	60.3	80.5	65.7	87.6	94.4	90.3	87.8	95.0	90.5
MMEA-cat ‡ [49]	62.4	84.5	70.2	64.1	86.9	72.3	72.5	91.4	79.3
GEEA ‡ [31]	76.1	94.6	82.7	75.5	95.3	82.7	77.6	96.2	84.4
MultiKE [20]	43.7	51.6	46.6	57.0	64.2	59.6	71.4	76.0	73.3
AttrE [19]	26.3	43.6	32.2	38.1	61.5	47.5	62.3	79.3	68.6
ICLEA † [36]	80.4	91.4	-	87.3	93.1	-	97.3	99.5	-
EVA ‡ [37]	75.2	89.5	80.4	73.7	89.0	79.1	73.1	90.9	79.2
SEU *† [40]	80.8	92.1	85.2	87.1	94.6	89.8	97.0	99.6	98.3
SelfKG *† [39]	73.8	86.0	77.1	81.5	91.3	84.9	94.2	98.8	97.2
UDCEA *† [41]	81.1	**92.2**	**85.5**	84.7	93.5	87.8	98.1	99.5	98.7
LCA-UEA (ours) †	**81.5**	91.5	85.1	**87.5**	**94.6**	**90.1**	**98.4**	**99.8**	**99.0**

The baselines marked with “*” are reproduced by using their source code, while the others are directly obtained from OpenEA [45] or their original papers. The baselines marked with “†” utilize pre-trained language models (e.g., LaBSE, FastText) to generate the initial embeddings of entity names, while those marked with “‡” incorporate image information. ’-’ indicates that the value is not reported in the original paper, so it is left blank. Additionally, we highlight the best performance results in the first two categories with underline and the best performance results among the unsupervised methods in **bold**. The notations in Table 4 and Table 5 are the same.

**Table 4:** Comparative results of LCA-UEA against 19 baselines on *WK31-15K*.

Datasets	${\mathrm{EN-FR}}_{V1}$			${\mathrm{EN-FR}}_{V2}$			${\mathrm{EN-DE}}_{V1}$			${\mathrm{EN-DE}}_{V2}$		
**Models**	**Hits@1**	**Hits@10**	**MRR**	**Hits@1**	**Hits@10**	**MRR**	**Hits@1**	**Hits@10**	**MRR**	**Hits@1**	**Hits@10**	**MRR**
MTransE [17]	24.6	56.2	35.0	24.4	52.8	34.0	30.9	61.2	40.9	19.6	43.3	27.7
BootEA [21]	50.3	78.6	59.7	66.1	90.8	74.7	67.1	86.6	73.7	84.9	94.5	88.3
RDGCN [23]	75.4	87.9	79.9	84.8	93.4	88.1	82.4	91.3	85.5	84.0	90.9	86.6
AliNet [24]	35.8	67.1	46.4	54.2	86.0	65.6	59.3	81.3	66.4	79.8	92.3	84.4
EMEA * [47]	63.8	91.1	73.3	85.5	98.3	90.5	75.1	94.1	81.9	87.6	98.0	94.4
RPR-RHGT [8]	90.9	96.6	93.0	94.9	98.5	96.3	92.1	97.2	94.0	93.8	97.8	95.3
STEA [46]	72.8	92.9	79.8	92.6	99.0	95.0	81.1	95.3	86.0	96.0	99.2	97.2
PEEA † [10]	76.6	92.0	80.4	88.9	98.2	92.5	78.7	95.4	84.5	95.7	99.0	97.0
RANM [9]	92.5	97.0	94.1	97.0	98.4	97.7	94.9	97.8	96.2	96.6	98.0	97.5
JAPE [18]	26.6	59.4	37.4	29.4	62.3	40.4	27.4	59.6	38.1	15.9	39.4	24.0
GCN-Align [48]	33.4	66.9	44.6	41.8	80.1	54.5	48.0	75.3	57.1	54.1	78.6	62.6
MRAEA * [27]	40.6	72.2	51.1	78.9	96.9	85.8	53.3	78.7	62.1	75.7	92.2	81.6
MHNA * [13]	92.9	96.4	94.5	96.1	98.4	97.2	94.1	97.4	95.5	95.7	98.2	96.9
SDEA *† [33]	97.1	98.9	97.8	97.6	99.2	98.1	97.2	99.0	97.9	97.7	99.4	98.3
MultiKE [20]	74.2	83.6	77.6	86.1	92.3	88.4	75.3	82.9	78.1	75.7	83.7	78.6
AttrE [19]	48.9	73.7	57.6	53.2	80.0	62.7	53.6	75.8	61.4	64.3	85.6	71.9
SEU *† [40]	97.5	99.3	98.6	95.1	99.3	96.5	97.2	99.0	97.9	95.4	97.9	96.3
SelfKG *† [39]	97.0	99.4	97.9	97.1	99.5	98.0	96.7	99.0	97.5	96.2	98.8	97.1
UDCEA *† [41]	97.6	99.4	98.2	97.8	99.1	98.2	96.6	98.6	97.4	94.8	98.0	96.0
LCA-UEA (ours) †	**98.6**	**99.8**	**99.1**	**98.8**	**99.7**	**99.2**	**97.7**	**99.4**	**98.3**	**96.8**	**98.8**	**97.5**

Due to the difficulty in acquiring image information for the *WK31-15K* dataset, we are unable to provide the experimental results for certain baselines that require image input, such as MMEA-cat [49], GEEA [31], and EVA [37].

**Table 5:** Comparative results of LCA-UEA against 15 baselines on *DWY100K*.

Datasets	DBP-WD			DBP-YG		
**Models**	**Hits@1**	**Hits@10**	**MRR**	**Hits@1**	**Hits@10**	**MRR**
MTransE [17]	28.1	52.0	36.3	25.2	49.3	33.4
BootEA [21]	74.8	89.8	80.1	76.1	89.4	80.8
AliNet [24]	69.0	90.8	76.6	78.6	94.3	84.1
EMEA * [47]	83.6	95.2	88.9	86.2	97.3	90.4
RPR-RHGT [8]	99.2	99.8	99.5	96.5	98.8	97.4
STEA * [46]	90.6	97.8	93.2	89.3	96.5	91.9
RANM [9]	99.3	99.8	99.5	97.2	99.4	98.0
JAPE [18]	31.8	58.9	41.1	23.6	48.4	32.0
GCN-Align [48]	50.6	77.2	57.7	59.7	83.8	68.6
MRAEA [27]	65.5	88.6	73.4	77.5	94.2	83.4
AttrGNN † [12]	96.0	98.8	97.2	99.8	99.9	99.9
MHNA * [13]	99.3	99.9	99.4	99.9	100.0	100.0
MultiKE [20]	91.8	96.2	93.5	88.0	95.3	90.6
SEU *† [40]	95.7	99.4	97.2	99.9	100.0	99.9
SelfKG *† [39]	98.0	99.8	98.9	99.8	100.0	99.9
LCA-UEA (ours) †	**98.3**	**99.8**	**98.9**	**100.0**	**100.0**	**100.0**

**Table 6:** Ablation study of LCA-UEA on *DBP-15K*. Results in **bold** are the best results.

Datasets	${\mathrm{ZH-EN}}_{\mathrm{DBP}}$			${\mathrm{JA-EN}}_{\mathrm{DBP}}$			${\mathrm{FR-EN}}_{\mathrm{DBP}}$		
**Models**	**Hits@1**	**Hits@10**	**MRR**	**Hits@1**	**Hits@10**	**MRR**	**Hits@1**	**Hits@10**	**MRR**
*w/o ECE+RRS*	79.4	89.8	83.1	85.6	93.3	88.4	98.1	99.6	98.7
*w/o ECE*	79.6	89.6	83.2	86.0	93.5	88.7	98.2	99.7	98.8
*w Cosine*	77.0	89.1	81.4	83.8	93.3	87.3	96.2	99.6	97.6
*w GAT*	74.3	90.2	80.1	81.6	93.7	86.1	97.7	99.6	98.5
*w GCN+GAT*	76.5	**91.8**	82.2	87.0	**95.6**	90.2	97.8	**99.9**	98.8
LCA-UEA (ours)	**81.5**	91.5	**85.1**	**87.5**	94.6	**90.1**	**98.4**	99.8	**99.0**

## Data Availability

Data are contained within the article.
